# Mitochondrial ferritin attenuates cerebral ischaemia/reperfusion injury by inhibiting ferroptosis

**DOI:** 10.1038/s41419-021-03725-5

**Published:** 2021-05-05

**Authors:** Peina Wang, Yanmei Cui, Qianqian Ren, Bingqi Yan, Yashuo Zhao, Peng Yu, Guofen Gao, Honglian Shi, Shiyang Chang, Yan-Zhong Chang

**Affiliations:** 1 Laboratory of Molecular Iron Metabolism, Key Laboratory of Animal Physiology, Biochemistry and Molecular Biology of Hebei Province, Ministry of Education Key Laboratory of Molecular and Cellular Biology, College of Life Science, Hebei Normal University, 050024 Shijiazhuang, Hebei Province China; 2 Scientific Research Center, Hebei University of Chinese Medicine, 050200 Shijiazhuang, Hebei Province China; 3 Department of Pharmacology and Toxicology, School of Pharmacy, University of Kansas, 1251 Wescoe Hall Drive, Malott Hall 5044, Lawrence, KS 66045 USA; 4 College of basic medicine, Hebei Medical University, 050017 Shijiazhuang, Hebei Province China

**Keywords:** Lipid peroxides, Iron, Cell death, Cell death in the nervous system, Stroke

## Abstract

Ischaemic stroke is becoming the most common cerebral disease in aging populations, but the underlying molecular mechanism of the disease has not yet been fully elucidated. Increasing evidence has indicated that an excess of iron contributes to brain damage in cerebral ischaemia/reperfusion (I/R) injury. Although mitochondrial ferritin (FtMt) plays a critical role in iron homeostasis, the molecular function of FtMt in I/R remains unknown. We herein report that FtMt levels are upregulated in the ischaemic brains of mice. Mice lacking FtMt experience more severe brain damage and neurological deficits, accompanied by typical molecular features of ferroptosis, including increased lipid peroxidation and disturbed glutathione (GSH) after cerebral I/R. Conversely, FtMt overexpression reverses these changes. Further investigation shows that *Ftmt* ablation promotes I/R-induced inflammation and hepcidin-mediated decreases in ferroportin1, thus markedly increasing total and chelatable iron. The elevated iron consequently facilitates ferroptosis in the brain of I/R. In brief, our results provide evidence that FtMt plays a critical role in protecting against cerebral I/R-induced ferroptosis and subsequent brain damage, thus providing a new potential target for the treatment/prevention of ischaemic stroke.

## Introduction

Stroke is a leading cause of disability and death worldwide^1,2^. An acute ischaemic stroke, accounting for approximately 87% of strokes, occurs as the result of vascular occlusion, leading to neuronal cell death and neurological deficits^3^. Clinically, the only approved therapy for stroke is to restore the blood flow either by pharmacological or mechanical thrombolysis^4^. However, only less than 10% of stroke patients are eligible for tissue plasminogen activator therapy, and half of those patients fail to demonstrate clinical improvement^5,6^. Reperfusion itself may cause secondary damage to neurons^7^. The mechanisms of ischaemia/reperfusion (I/R) injury are multifaceted, including oxidative stress, inflammation and excitotoxicity, among others^8^. Considerable evidence has emerged in recent years to indicate that iron is a risk factor in the development of cerebral I/R^9,10^. The iron content is increased in ischaemic brains^10,11^. Iron-overloaded animals are more affected by middle cerebral artery occlusion (MCAO)^12^, whereas iron chelation or depletion reduces I/R-induced brain injury^13,14^. Moreover, in patients with acute ischaemic stroke, an elevated serum iron storage correlates with a higher risk of poor clinical outcome^15^. The underlying mechanisms of cerebral I/R-mediated iron overload and iron toxicity-induced neuronal cell death remain unclear.

Ferroptosis is a novel iron-dependent form of regulated cell death (RCD) that is genetically, morphologically, and biochemically distinct from apoptosis, autophagy and necroptosis^16^. The biochemical mechanism underlying ferroptosis is iron-dependent formation of lipid reactive oxygen species (L-ROS) combined with depletion of glutathione (GSH) or inactivation of the lipid repair enzyme GSH peroxidase 4 (GPX4)^16,17^. Aberrant accumulation of L-ROS, resulting from iron-catalyzed peroxidation of polyunsaturated fatty acids (PUFAs), leads to membrane rupture and subsequent cell death. L-ROS inhibitors or iron chelators can suppress or reverse this lethal process^18,19^. Ferroptosis has been implicated in several pathophysiological processes associated with degenerative diseases, carcinogenesis, liver damage and kidney ischaemia/reperfusion injury^19,20^. In addition, recent studies have indicated that ferrostatin-1 (Fer-1), an inhibitor of ferroptosis, can alleviate cerebral I/R-induced brain damage in mice^11,21^. However, the relevance of ferroptosis in ischaemic stroke is still poorly understood and remains enigmatic.

Mitochondrial ferritin (FtMt) is a key mitochondrial iron storage protein with high homology to the heavy chain of cytosolic ferritin (FtH)^22–24^. FtMt has ferroxidase activity, catalysing the conversion of Fe^2+^ to the ferric form for storage in the FtMt spherical shell, which can accommodate up to 4000 iron atoms. Moreover, the expression of FtMt is tissue-specific, showing high levels in cells with high oxygen consumption, such as those of the testes and central nervous system, while no expression has been observed in the liver and spleen, the main iron storage tissues^25^. These properties suggest that the major role of FtMt is to protect cells in specific tissues from iron-dependent oxidative damage rather than being directly related to cellular iron levels^26–28^. We and others have indicated that FtMt-deficient mice do not show any evident phenotypes or iron-related disorders under baseline feeding conditions but that FtMt exerts significant protective effects under pathological conditions, such as in Alzheimer’s disease and Parkinson’s disease^28–31^. In addition, our previous study has shown that FtMt can inhibit erastin-induced ferroptosis in vitro^32^. More interestingly, we recently found that FtMt is a hypoxia-inducible factor 1α (HIF-1α) target gene that is upregulated under hypoxia, indicating that FtMt may be a potential therapeutic target in situations of hypoxic challenge, such as ischaemic stroke^33^. Therefore, we hypothesized that FtMt may be protective in I/R by regulating iron homeostasis and ferroptosis. However, any possible role of FtMt in cerebral I/R injury is still unknown.

Inspired by these findings, we investigated the role of FtMt and FtMt-associated ferroptosis in cerebral I/R in this study. Our data suggest that FtMt overexpression attenuates I/R-induced brain damage and neurological deficits. We also provide evidence that the ferroptosis pathway is activated in cerebral I/R, including upregulation of acyl-CoA synthetase long-chain family member 4 (ACSL4), reductions in glutathione levels and other changes. The deletion of FtMt promotes I/R-induced inflammation and hepcidin-mediated decreases in ferroportin1 (FPN1), thus markedly increasing total and chelatable iron. The excess free iron in FtMt-deficient mice in turn exacerbates L-ROS-induced ferroptosis in the brains of I/R mice. Our results provide new insights relevant to the treatment and/or prevention of cerebral I/R injury.

## Results

### Upregulation of FtMt in the I/R brains of mice

**Fig. 1:**
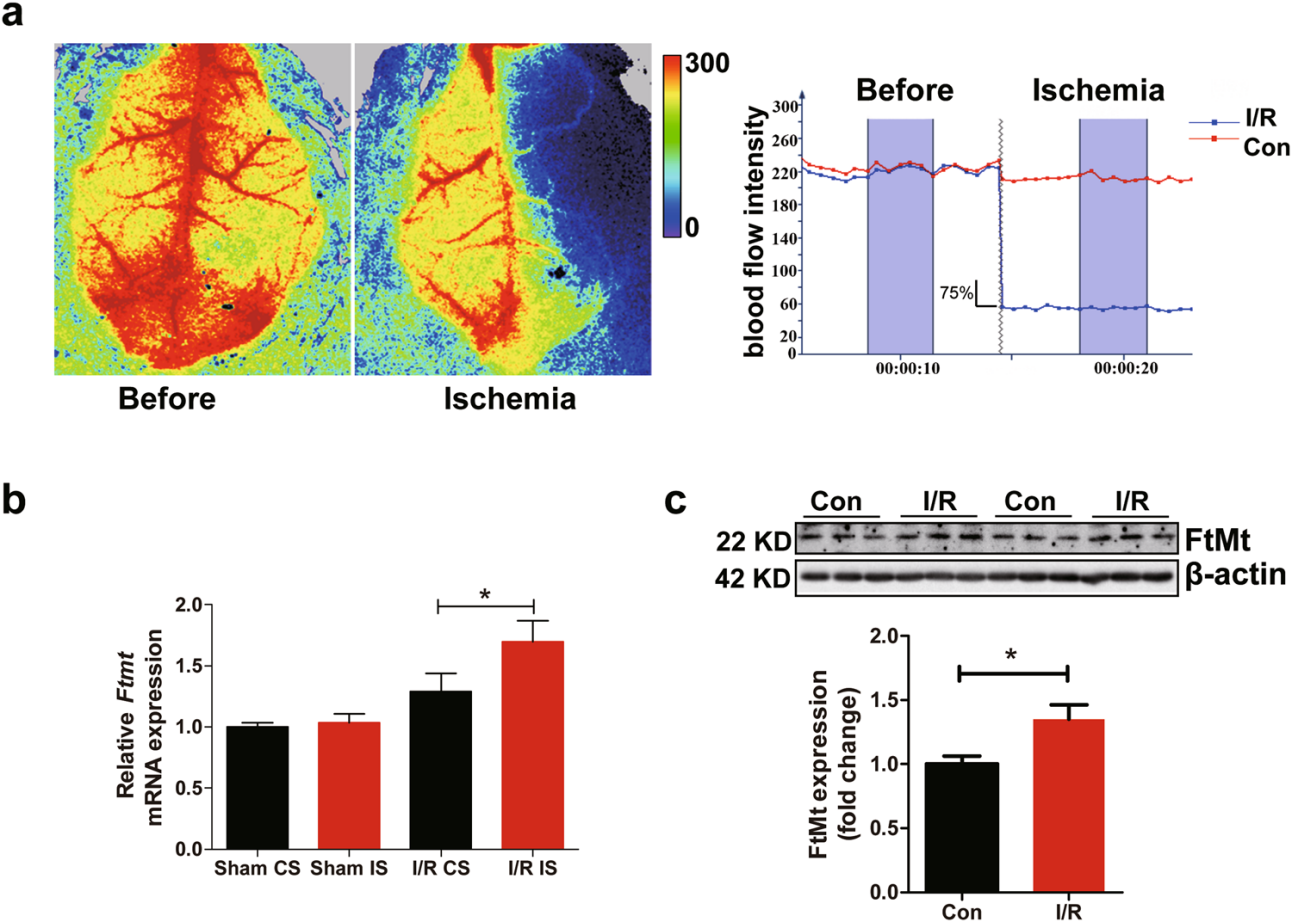
Upregulation of FtMt in the I/R brains of mice. **a** Ischaemic stroke was induced by MCAO for 90 min with a subsequent 24 h reperfusion period. Blood flow was detected by laser Doppler flowmetry during MCAO. **b** Relative mRNA expression of *Ftmt* in operated mice or sham-operated controls. The mRNA levels were normalized to β-actin mRNA levels and are expressed relative to the mean value in the sham control side. CS, control side; IS, injured side. **c** Western blot analyses showing the induction of FtMt in the penumbra after 24 h of reperfusion. The results are normalized to the β-actin levels. I/R, penumbral area in the cortex of the ipsilateral hemisphere; Con, same area in the penumbra in the cortex of the contralateral hemisphere. The results are presented as the mean ± SEM (*n* = 6). *P < 0.05.

To examine whether FtMt contributes to cerebral I/R injury, we first measured the expression of FtMt in I/R brains. We performed MCAO surgery on mice. Focal cerebral blood flow was measured by laser Doppler flowmetry. The mice whose blood flow in the right hemisphere was reduced by more than 75% (Fig. 1a) were selected for examination. The mRNA expression of FtMt was significantly increased on the I/R side compared with the control side or the brains of sham-operated mice (Fig. 1b). We also detected significant upregulation of FtMt protein expression on the I/R side (Fig. 1c). The observation that FtMt levels are markedly increased under I/R conditions suggests that FtMt may participate in brain I/R injury. The penumbra has been considered a therapeutic target in stroke patients. In our study, all the samples were prepared from the penumbra of the cortex (I/R side) or the same area of the control side (Con side).

### Loss of FtMt exacerbates cerebral I/R-induced brain damage

**Fig. 2:**
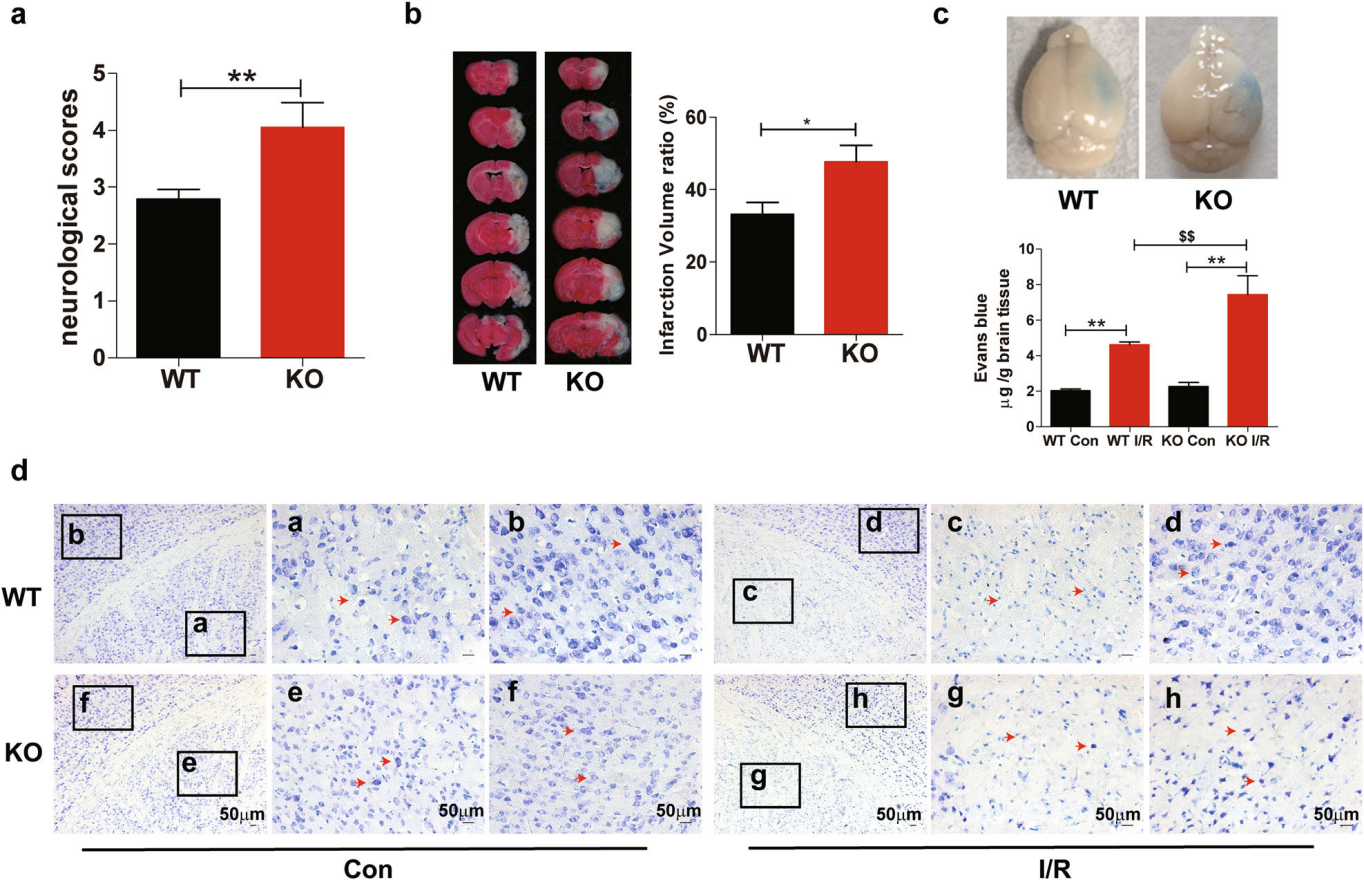
Loss of FtMt exacerbates cerebral I/R-induced brain damage. Wild-type and FtMt-knockout mice were subjected to MCAO for 90 min and subsequent reperfusion for 24 h. **a** After 24 h of reperfusion, the neurologic deficit scores of wild-type and *Ftmt*-knockout mice were compared (*n* = 15). **b** Infarct volumes were compared between wild-type (*n* = 5) and *Ftmt*-knockout mice (*n* = 6) by TTC staining of coronal sections. **c** Representative images (upper panels) and quantification (lower panels) of Evans blue dye extravasation in wild-type or *Ftmt*-knockout mice at 24 h after I/R (*n* = 5). **d** Nissl staining was performed after 90 min of MCAO and 24 h of reperfusion, and the I/R sides and control (Con) sides of wild-type and *Ftmt*-knockout mice were compared. The red arrows denote the altered Nissl bodies and degenerated neurons (*n* = 3). WT, wild-type mice; KO, *Ftmt*-knockout mice. The results are presented as the mean ± SEM. *^/$^*P* < 0.05, **^/$$^*P* < 0.01.

To explore the role of FtMt in brain I/R injury, we subjected wild-type and *Ftmt-*knockout mice to MCAO. Neurologic assessment revealed that ablation of FtMt aggravated I/R-induced neurologic deficits (Fig. 2a). The infarct volumes were analyzed by TTC staining after 24 h of reperfusion, *Ftmt*-knockout mice showed significantly higher infarct volumes than wild-type mice (Fig. 2b). Evans blue extravasation assays indicated that FtMt knockout exacerbated I/R-induced blood–brain barrier (BBB) leakage at 24 h after reperfusion (Fig. 2c). We also performed Nissl staining to evaluate the effect of FtMt deficiency on I/R-induced morphological alterations. As shown in Fig. 2d, on the control sides of both wild-type and *Ftmt*-knockout mice, the cells were stained evenly in the cortex and striatum and were large with an abundant cytoplasmic compartment. In contrast, the neurons on the I/R sides were sparsely distributed and exhibited shrunken cell bodies. This effect was more pronounced in the FtMt-deficient mice, which showed severe neuronal damage. These data indicate that FtMt deletion exacerbates I/R-induced brain damage.

### FtMt deletion aggravates cerebral I/R-induced ferroptosis

**Fig. 3:**
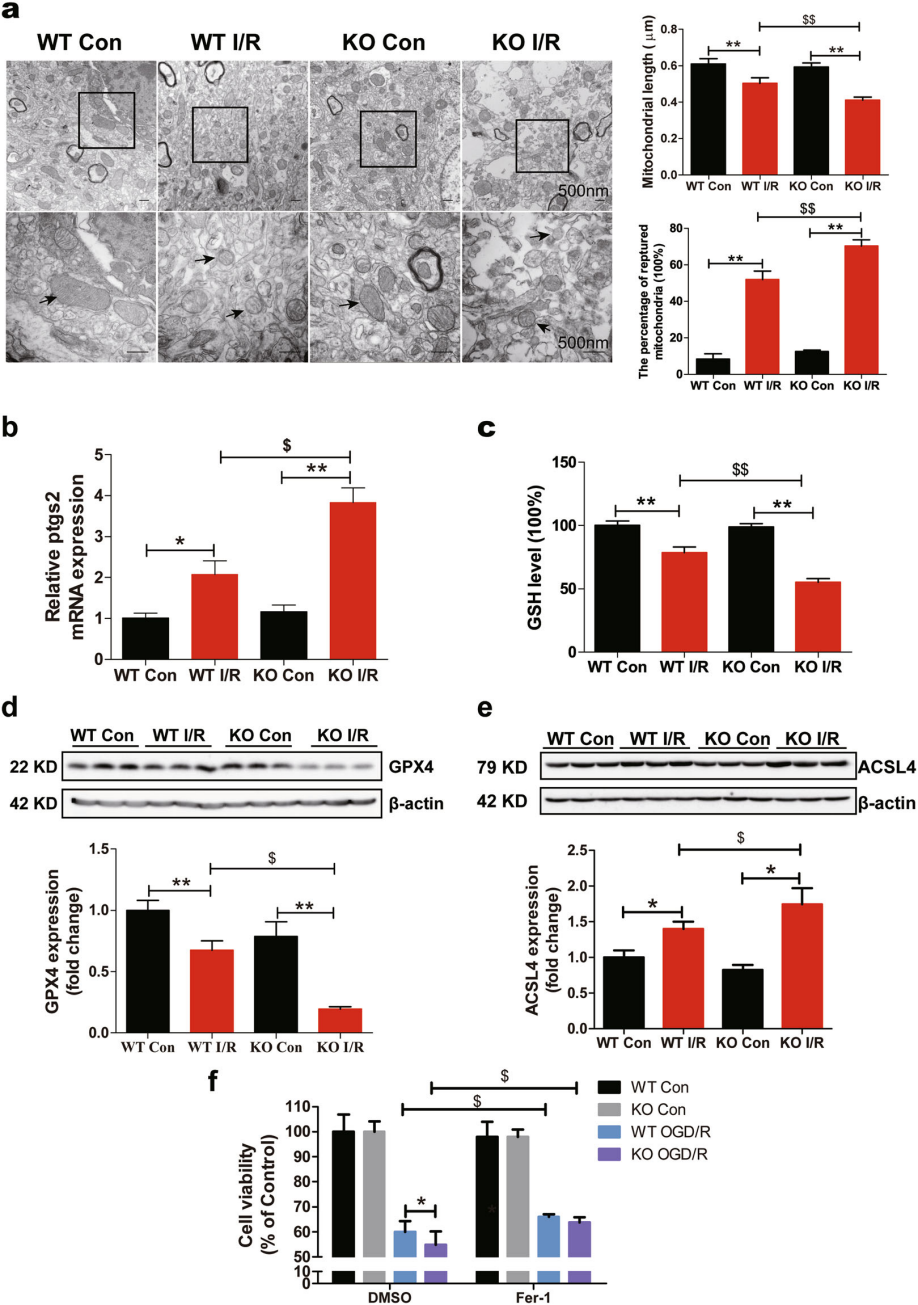
FtMt deletion aggravates cerebral I/R-induced ferroptosis. Wild-type (WT) and *Ftmt*-knockout mice were subjected to MCAO (90 min) and subsequent reperfusion (24 h). **a** Transmission electron microscopy of cells in the control (Con) and I/R groups. Mitochondrial length and the percentage of ruptured mitochondria in different groups were measured and averaged (*n* = 3). **b** The relative mRNA expression of *Ptgs2* was measured in Con and I/R tissues. The mRNA levels were normalized to β-actin mRNA levels and are expressed relative to the mean value in the WT Con group (*n* = 6). **c** The GSH content was measured in mice. The data are expressed relative to the mean value in the WT Con group (*n* = 5). Western blot analysis of (**d**) GPX4 and (**e**) ACSL4 at 24 h after reperfusion. The data are expressed relative to the mean value in the WT Con group (*n* = 5). **f** Primary neurons were pre-treated with 0.05 μM Fer-1 for 4 h and then subjected to OGD/R. Cell viability was measured using an MTT assay (*n* = 6). The results are presented as the mean ± SEM. *^/$^*P* < 0.05, **^/$$^*P* < 0.01.

To explore how *Ftmt* deletion aggravates neuronal damage and whether ferroptosis is activated in the brain during I/R, we detected the effects of *Ftmt* deletion on ferroptosis in I/R. *Ftmt* knockout exacerbated I/R-induced mitochondrial damage. Compared to the control side, the I/R side in both the wild-type and *Ftmt-*knockout mice had smaller, ruptured mitochondria (Fig. 3a), these morphological features are characteristic of ferroptosis^18,20^. *Ftmt* knockout caused significant reduction in mitochondrial length and increased numbers of ruptured mitochondria on the injured side compared with wild-type mice (Fig. 3a). The mRNA expression of *Ptgs2*, a core biomarker of ferroptosis, was significantly increased on the I/R side, and the increase was more apparent in *Ftmt*-knockout mice (Fig. 3b). Additionally, the levels of two negative regulators, GSH and glutathione peroxidase 4 (GPX4), were decreased after 24 h of reperfusion, FtMt deficiency exacerbated the blockade of this antioxidant system (Fig. 3c, d). The expression of the positive regulator ACSL4, which facilitates the formation of L-ROS, was induced in the ischaemic brain, with *Ftmt* deletion leading to a greater increase (Fig. 3e). To further confirm the involvement of ferroptosis in I/R, SH-SY5Y cells were treated with Fer-1 in the OGD/R (oxygen-glucose deprivation followed by reoxygenation) system. Fer-1 significantly attenuated OGD/R-induced cell loss at 0.05 and 0.1 μM (Supplementary Figure 1A). We then prepared primary cortical neurons from wild-type and *Ftmt*-knockout mice and exposed the cells to OGD/R plus 0.05 μM Fer-1. Our results showed that Fer-1 elicited a protective effect on primary cultured cells exposed to OGD/R (Fig. 3f), which is consistent with our in vivo findings. These results provide morphological and molecular evidence that neurons in the I/R brain undergo ferroptosis, which is exacerbated by the absence of FtMt.

### Ablation of FtMt promotes lipid peroxidation in I/R brains

**Fig. 4:**
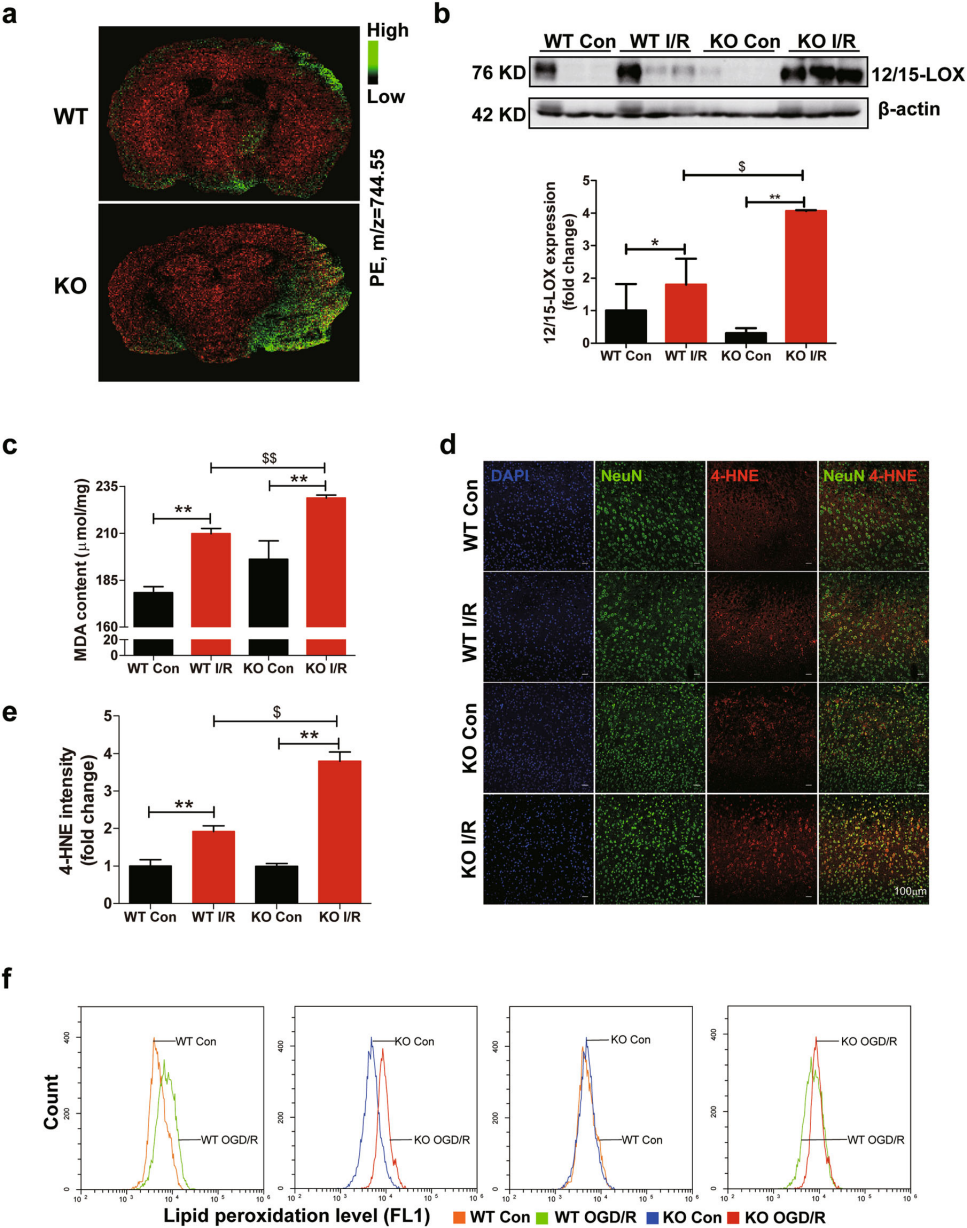
Ablation of FtMt promotes lipid peroxidation in I/R brains. **a** MSI heat maps for the distribution of PE (*m/z* = 744.55, green) in wild-type (WT) and *Ftmt*-knockout (KO) mouse brains after ischaemic stroke. **b** Western blot analysis of 12/15-LOX in WT and KO mice after MCAO (90 min) and subsequent reperfusion (24 h) (*n* = 6). **c** The mouse brain MDA content was measured. The data are expressed relative to the mean value in the WT control (Con) group (*n* = 5). **d** Representative immunofluorescence images of 4-HNE (red) and Neun (green). **e** Quantification of 4-HNE fluorescence intensity (*n* = 3). **f** Flow cytometry analysis of OGD/R-induced C11-BODIPY (581/591) oxidation in primary cultured WT and KO neurons. The data shown are representative of two independently performed experiments. The results are presented as the mean ± SEM. */^$^*P* < 0.05, **^/$$^*P* < 0.01.

The biochemical mechanism underlying ferroptosis is the iron-dependent formation of L-ROS^34^. Lipidomic studies have suggested that phosphatidylethanolamines (PEs) are key phospholipids that undergo oxidation and drive cells towards ferroptotic death^35^. Mass spectrometry imaging was used to analyze the distribution of PEs in brains after ischaemic stroke. As shown in Fig. 4a, PE levels were increased on the I/R side in both knockout and wild-type mouse brains, however, PEs were much more abundant in knockout stroke samples. 12/15-lipoxygenase (12/15-LOX) is an iron-dependent enzyme that oxidizes phospholipids and plays a key role in ferroptosis^36,37^. We found that the expression of 12/15-LOX was higher in the *Ftmt*-knockout I/R group than in the wild-type I/R group (Fig. 4b). Additionally, both the MDA (Malondialdehyde) content and the expression of 4-HNE (4-hydroxynonenal) were increased after I/R, with *Ftmt* deletion leading to greater increases in these two features (Fig. 4c–e). To further verify the role of FtMt in L-ROS formation in the cerebral I/R model, we prepared primary cortical neurons from wild-type and *Ftmt*-knockout mice and exposed the cells to OGD/R. Assessment of the production of L-ROS by the C11-BODIPY (581/591) method revealed that FtMt deficiency aggravated OGD/R-induced L-ROS accumulation in the cultured neurons (Fig. 4f). Taken together, our results indicate that FtMt deletion potentiates I/R-induced L-ROS formation and ferroptosis in mice.

### Deletion of FtMt leads to an elevation in iron after cerebral I/R

**Fig. 5:**
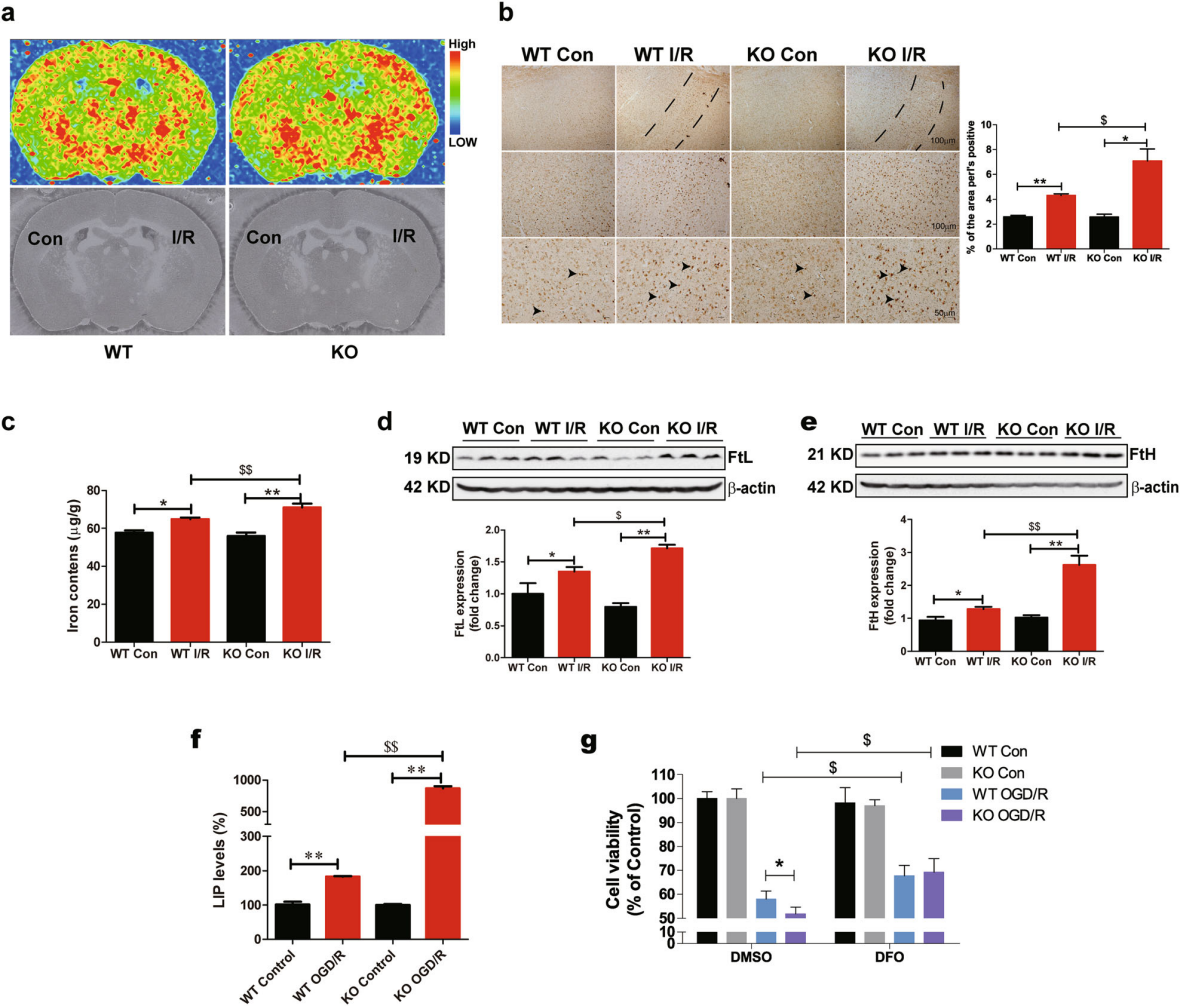
Deletion of FtMt leads to an elevation in iron after cerebral I/R. **a** Distribution of iron in the mouse brain after I/R. The upper images show the mapping results with μ-XRF, and the iron levels in different brain regions are indicated by different colours. Blue indicates the lowest iron level, and red indicates the highest iron level, as shown in the illustration. The lower images show the morphology of the brain sections corresponding to the upper images (*n* = 3). **b** Content and distribution of iron as determined by DAB-enhanced Perl’s staining and quantification of the area of Perl’s staining in brain sections from mice of different groups (*n* = 3). The dotted zone indicates the penumbra. The arrowheads indicate positive staining of iron. **c** Iron content as determined by ICP-MS in wild-type and *Ftmt*-knockout mice 24 h after MCAO/reperfusion (*n* = 6). Western blot analysis of the iron storage proteins (**d**) FtL and (**e**) FtH (*n* = 5). **f** Primary cultured wild-type and *Ftmt*-knockout neurons were subjected to OGD for 5 h and reperfusion for 18 h, and the LIP levels were determined (*n* = 4). **g** Primary cultured wild-type and *Ftmt*-knockout neurons were subjected to OGD treatment for 5 h and then exposed to normal medium with 0.05 μm DFO for 18 h. Cell viability was determined using an MTT assay (*n* = 6). The results are presented as the mean ± SEM. *^/$^*P* < 0.05, **^/$$^*P* < 0.01.

As mentioned above, iron is the key factor in the pathological process of I/R. We hypothesized that FtMt may participate in I/R-induced ferroptosis and brain injury by affecting free iron accumulation. We used µ-XRF (micro-X-ray fluorescence) to directly detect the iron distributions in the brains of wild-type and *Ftmt*-knockout mice after I/R. Our data indicated that iron content was increased in the penumbra and that *Ftmt* gene knockout increased total iron accumulation compared with the wild-type I/R group (Fig. 5a). Perl’s stained sections revealed the same results (Fig. 5b). ICP-MS analysis showed that iron content was increased in the damaged areas of the I/R brain and was higher in the *Ftmt-*knockout I/R group than in the wild-type I/R group (Fig. 5c). The zinc content was unchanged (Supplementary Fig. 1B). We then detected the expression of FtL and FtH, subunits of the cytosolic iron storage protein ferritin. The levels of FtL and FtH were increased, and the increases were more noticeable in knockout mice (Fig. 5d, e), which also indicated that iron overload was more significant in the brains of *Ftmt*-knockout mice. In general, the toxicity of iron is directly related to the size of the labile iron pool (LIP). We therefore examined the effect of FtMt deficiency on LIP levels under I/R by estimating LIP levels in primary cultured wild-type and *Ftmt*-knockout neurons with or without OGD/R treatment. As shown in Fig. 5f, LIP levels were significantly increased in the *Ftmt-*knockout OGD/R group compared with the wild-type OGD/R group.

To verify that the increase in free iron in *Ftmt*-knockout mice exacerbated I/R-induced brain damage, we applied an iron chelator to the OGD/R-induced cell death model. SH-SY5Y cells were subjected to OGD/R treatment with different concentrations of the iron chelator DFO (deferoxamine). Treatment with DFO significantly attenuated OGD/R-induced cell loss at concentrations of 0.05 μM, 0.1 μM and 1 μM (Supplementary Fig. 1C). We then repeated this experiment in primary cultured wild-type and *Ftmt*-knockout neurons. The cells lacking FtMt exhibited exacerbated OGD/R-induced cell death that was almost completely reversed to the levels in wild-type neurons when 0.05 μM DFO was included during reperfusion (Fig. 5g).

### FtMt ablation promotes hepcidin expression and FPN1 degradation in I/R

**Fig. 6:**
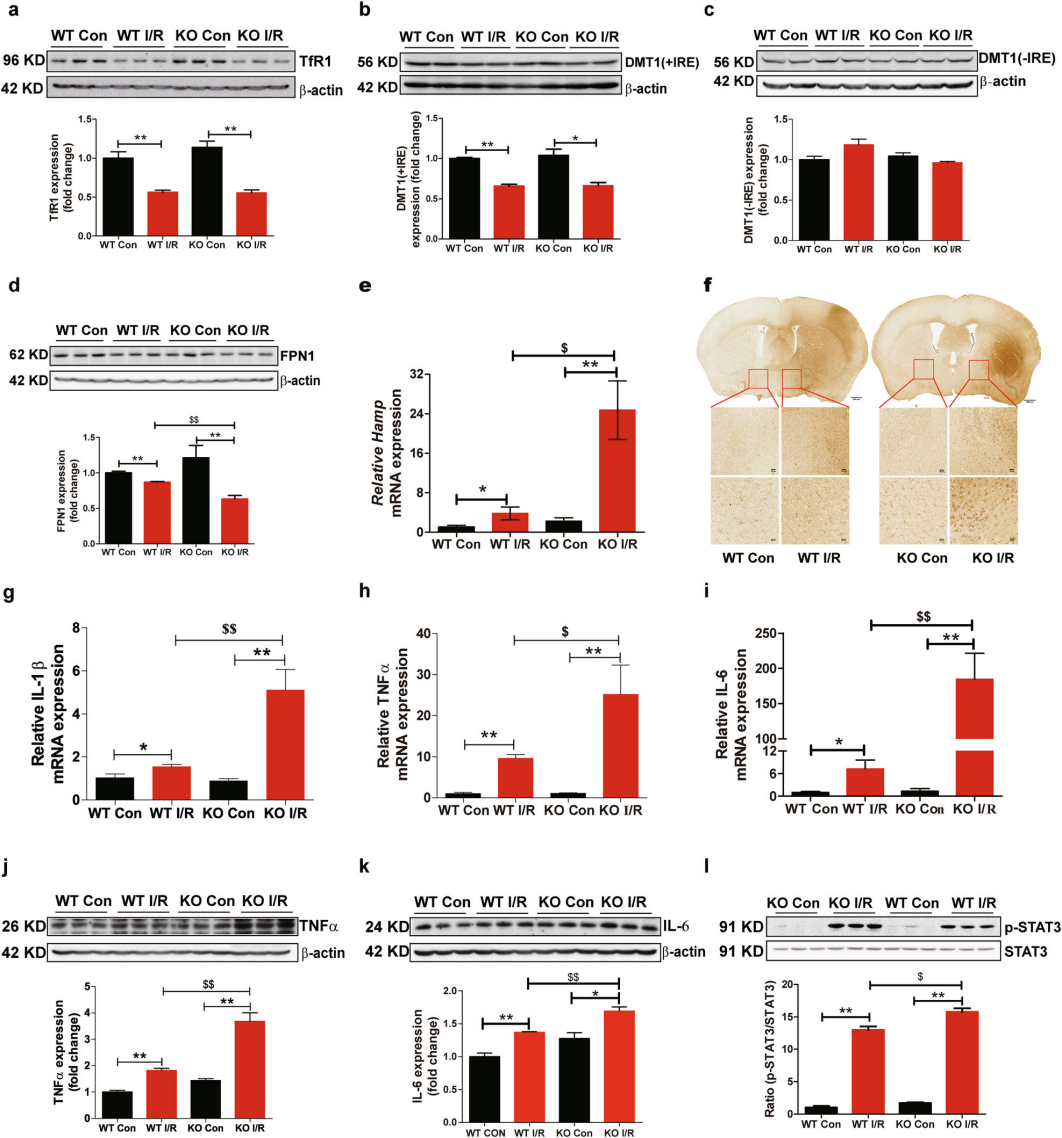
FtMt ablation promotes hepcidin expression and FPN1 degradation in I/R. Western blot analysis of the iron uptake proteins (**a**) TfR1, (**b**) DMT1(+IRE), and (**c**) DMT1(-IRE) and the iron export protein (**d**) FPN1 in wild-type (WT) and *Ftmt*-knockout mice (*n* = 5). **e** The relative mRNA expression of hepcidin (*Hamp*) was measured in the control (Con) and I/R groups. The mRNA levels were normalized to β-actin mRNA levels and are expressed relative to the mean value in the WT Con group (*n* = 6). **f** Brain sections derived from WT and *Ftmt-*knockout mice after cerebral I/R were immunostained for Iba-1 to assess the activation of microglia. The relative mRNA levels of (**g**) IL-1β, (**h**) TNFα and (**i**) IL-6 were measured in the Con and I/R sides of mouse brains. The mRNA levels were normalized to β-actin mRNA levels and are expressed relative to the mean value in the WT Con group (*n* = 5). The expression of (***j***) TNFα, (*n* = 5) (**k**) IL-6, (*n* = 5) (**l**) p-STAT3 and STAT3 (*n* = 6) was assessed by Western blot analysis. The results are presented as the mean ± SEM. *^/$^*P* < 0.05, **^/$$^*P* < 0.01.

To further clarify the mechanisms that mediate the regulation of iron homeostasis in *Ftmt*-knockout mice after I/R, iron metabolism-related proteins were examined. The levels of the iron uptake proteins TfR1 (transferrin receptor 1) and DMT1(+IRE) (divalent metal transporter 1) were decreased (Fig. 6a, b), whereas the levels of DMT1(-IRE) were similar between the different groups (Fig. 6c). However, we did not find significant differences in the levels of these proteins between wild-type mice and *Ftmt*-knockout mice. Interestingly, we observed decreased levels of FPN1, the only cellular iron exporter, after cerebral I/R, and deletion of FtMt promoted the decreases in FPN1 levels (Fig. 6d). These results indicated that the significant reduction in FPN1 expression in *Ftmt*-knockout mice increased the severity of cellular iron overload in I/R.

Hepcidin is an iron regulatory hormone that can bind to FPN1 to induce its degradation or directly inhibit its iron export activity, thereby decreasing iron efflux^38^. We have previously reported that I/R-induced inflammation causes upregulation of hepcidin via the IL-6 -STAT3 pathway^10,39^. To confirm the mechanism of the decline in FPN1 expression in I/R, we further detected the expression of hepcidin. As shown in Fig. 6e, the level of hepcidin on the I/R side was significantly increased, with the level in the *Ftmt*-knockout I/R group approximately 4-fold higher than that in the wild-type I/R group. The inflammatory response is a major culprit for secondary damage after reperfusion, and inflammation can also induce hepcidin expression. Therefore, we next examined the activation of microglia, the major contributors to the inflammatory response in the CNS, as well as the expression levels of inflammatory factors. As shown in Fig. 6f, the microglia in the penumbras of *Ftmt*-knockout mice exhibited the characteristic morphology of activated cells and displayed enlarged cell bodies. Real-time RT-PCR analysis revealed that FtMt deficiency increased the I/R-induced mRNA expression of pro-inflammatory cytokines (TNF-α, IL-1β and IL-6) (Fig. 6g–i). At the same time, western blots analysis shown that the levels of TNF-α, IL-6 and phosphorylated STAT3 (p-STAT3) were also increased (Fig. 6j–l). These results demonstrate that FtMt deficiency promotes the I/R-induced inflammatory response and hepcidin expression. Upregulation of hepcidin leads to degradation of FPN1 and results in iron accumulation in neuronal cells.

### FtMt overexpression attenuates iron-mediated ferroptosis and brain damage in cerebral I/R

**Fig. 7:**
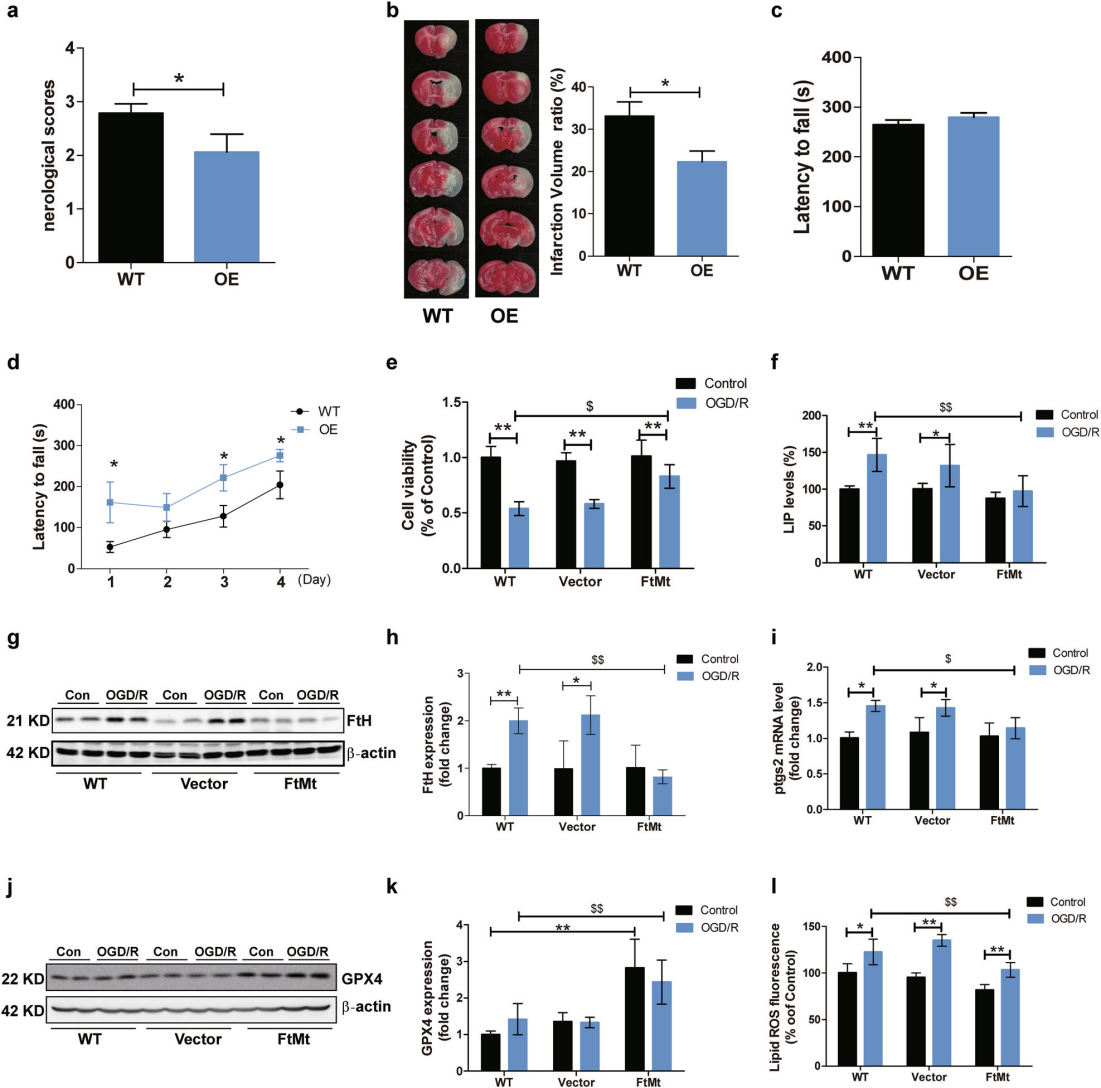
FtMt overexpression attenuates iron-mediated ferroptosis and brain damage in cerebral I/R. Wild-type and FtMt-overexpressing mice were subjected to I/R surgery. **a** Neurologic deficit scores (*n* = 15) and (**b**) infarct volumes (*n* = 5) were measured. Motor coordination was measured by the rotarod treadmill test as described in the “Materials and Methods” section. **c** Performance of wild-type and FtMt-overexpressing mice on the last day of training before surgery. **d** Performance of wild-type and FtMt-overexpressing mice after cerebral I/R (*n* = 10). WT, wild-type mice; OE, FtMt-overexpressing mice. Wild-type (WT) SH-SY5Y cells, empty vector transfectants (vector) and FtMt-overexpressing transfectants (FtMt) were subjected to OGD/R treatment. **e** Cell viability, (**f**) LIP levels, and (**g**) FtH expression were measured after OGD/R. **h** Quantification of FtH levels (*n* = 4). **i** Relative mRNA expression of *Ptgs2* (*n* = 4). The mRNA levels were normalized to β-actin mRNA levels and are expressed relative to the mean value of the WT control (Con) group. **j** Expression of GPX4 in three types of cells after OGD/R. **k** Quantification of GPX4 levels (*n* = 5). **l** L-ROS fluorescence (C11-BODIPY [581/591] oxidation) was measured in different groups. The results are presented as the mean ± SEM. */$*P* < 0.05, **/$$*P* < 0.01.

To further confirm the protective effects of FtMt during cerebral I/R, we performed MCAO surgery on FtMt-overexpressing mice. The neurologic deficit scores and infarct volume were both significantly decreased in the FtMt-overexpressing mice (Fig. 7a, b). In addition, we used the rotarod test to examine the effects of FtMt overexpression on the functional impairments elicited by I/R injury. The performances of wild-type and FtMt-overexpressing mice were similar before surgery (Fig. 7c), however, FtMt overexpression significantly attenuated the functional impairments after MCAO and reperfusion (Fig. 7d). Then, we further evaluated whether FtMt overexpression could inhibit free iron accumulation and ferroptosis in an OGD/R model. SH-SY5Y cells (the WT group), stable FtMt-expressing SH-SY5Y cells (the FtMt group) and pcDNA3.1(-) empty vector-transfected cells (the Vector group) were generated as previously described^29^ and then subjected to OGD/R treatment. We found that the cell viability of the FtMt overexpression group was significantly greater after OGD/R treatment than that of the other two groups (Fig. 7e). FtMt overexpression markedly diminished OGD/R-induced free iron accumulation and FtH upregulation (Fig. 7f–h). In addition, the levels of key biomarkers of ferroptosis, including *Ptgs2* expression, GPX4 expression and L-ROS content, were all improved in the FtMt overexpression group after OGD/R (Fig. 7i–l).

## Discussion

**Fig. 8:**
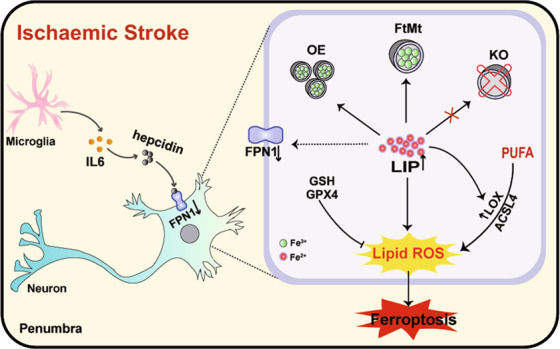
Schematic representation of the proposed neuroprotective mechanism of FtMt following cerebral I/R. Ischaemic stroke causes inflammation and iron dysregulation in neurons, which results in increases in intracellular LIP levels. Free iron may promote the generation of L-ROS and subsequent ferroptosis in penumbral cells. FtMt can withdraw iron from the cytoplasmic pool and inhibit ferroptosis, thus in turn attenuating brain damage in I/R.

In the present study, we show that mitochondrial ferritin plays an essential role in maintaining intracellular iron distribution and ROS production; thus, FtMt can prevent iron-induced ferroptosis and brain damage in ischaemic stroke, as shown in Fig. 8. Loss of FtMt facilitates free iron accumulation, increasing the generation of lipid ROS and leading to neuronal cell ferroptosis in cerebral I/R. In addition, the absence of FtMt promotes I/R-induced microglial activation and inflammation, which in turn exacerbates brain iron deposition by hepcidin-mediated FPN1 inactivation.

Iron, the most abundant transitional metal in the brain, is essential for normal neuronal function and activity. The synthesis of several neurotransmitters is iron-dependent and proper functioning of enzymatic systems that regulate cellular energy^40^. However, iron overload is involved in the formation of free radicals and produces oxidative damage to neuronal cells. In this study, we obtained in situ iron distribution mapping evidence using μ-XRF microspectroscopy that cerebral I/R leads to brain iron accumulation on the I/R side. In response, the cytosolic iron storage protein FtH and mitochondrial ferritin are upregulated to sequester excess iron (Fig. 5; Fig. 1). What is the mechanism by which iron is abnormally increased in the injured area after I/R? The inflammatory response is a major culprit and feature for the degree of I/R damage and is an important factor affecting iron accumulation in I/R^41^. A lack of oxygen and glucose could directly induce microglia activation and thereafter neuroinflammation^42^. In addition, evidence has shown that damaged or dead neuronal cells and excess ROS can activate an inflammatory response and upregulate pro-inflammatory gene expression after I/R injury^42,43^. We observed upregulation of IL-6, strong activation of microglia and accompanying BBB disruption during ischaemic stroke (Fig. 6; Fig. 2c). Subsequently, inflammation caused further upregulation of hepcidin through the IL-6-STAT3 pathway (Fig. 6e). The increased inhibitory effect of hepcidin on FPN1 aggravated I/R-induced iron overload in mice. Thus, excessive free iron, oxidative stress and inflammation form a vicious feedback cycle in ischaemic stroke, and the absence of FtMt exacerbates this cycle. On the one hand, without the protection of FtMt, the LIP level is markedly increased after I/R, resulting in detrimental accumulation of L-ROS and neuronal cell death, thus exacerbating the subsequent inflammatory response; on the other hand, the upregulation of inflammatory factors in turn affects brain iron homeostasis by hepcidin, causing further free iron accumulation in the brains of *Ftmt*-knockout mice. Therefore, FtMt plays a very important role in inhibiting the abnormal increase of iron in the brain after I/R injury.

Ferroptosis is a newly identified iron-dependent form of RCD driven by excessive accumulation of L-ROS. Aberrant lipid peroxidation in ferroptosis can take place via two approaches in the presence of iron: a nonenzymatic free radical chain reaction involving Fenton chemistry and enzymatic processes, such as those involving the iron-dependent enzyme LOX^44^. The free iron in the LIP is the source of the Fenton reaction that generates free radicals that are able to cause peroxidation of PUFAs. In addition, several lipid oxidation enzymes are iron-containing or iron-dependent enzymes that can facilitate L-ROS generation in ferroptosis, and LOX enzymes have been found to be the most important for ferroptosis^16,44^. Therefore, cellular iron overload is a key factor in ferroptosis initiation. It has been found that a high-iron diet can cause ferroptosis in the liver^20^. Furthermore, silencing TFRC, the gene encoding TfR1, to decrease iron content can inhibit erastin-induced ferroptosis, while upregulation of haem oxygenase-1 can accelerate ferroptosis by liberating iron from cellular haem sources^19,45^. In the present study, we found that increases in iron combined with accumulation of ROS exacerbate I/R-induced ferroptosis in *Ftmt*-knockout mice. We also provide the first evidence that I/R-treated neuronal cells exhibit hallmark features of ferroptosis, including shrunken mitochondria, overexpression of *Ptgs2*, and downregulation of GPX4, and that FtMt deletion aggravates these changes (Fig. 3). Previous studies have confirmed that PEs upregulates *ptgs2* in cells undergoing ferroptosis and *ptgs2* upregulation is a suitable and downstream maker of ferroptosis^46–48^. The expression of ACSL4 and 12/15-LOX, which catalyze lipid peroxidation, was increased in the mouse brain after I/R. Deletion of FtMt promoted the activation of these molecules (Fig. 3; Fig. 4). Fer-1 treatment or iron chelation with DFO reduced OGD/R-induced injury, consistent with the findings of previous in vivo studies^11,49,50^. In this study, we demonstrate that FtMt deficiency can induce iron overload, which exacerbates L-ROS production and ferroptosis in I/R; conversely, overexpression of FtMt affords protection to neuronal cells after ischaemic stroke.

Following a stroke, the supply of glucose and energy to neurons is disrupted, resulting in irreversible necrotic cell death and the formation of an infarct. Cells in the penumbra remain metabolically active and are usually able to die in a regulated manner by RCD^7^. Although other RCD pathways, such as apoptosis and necroptosis, have been explored in the MCAO model and numerous drug candidates that inhibit these pathways have demonstrated neuroprotection in rodent models of stroke^1,51^, almost all these strategies failed to translate to the clinic^52^. Hence, it is vital to further understand the mechanisms of I/R injury, and there may be other cell death pathways that play a crucial role in neuronal cell fate decisions in the penumbra. In this study, we provide the evidence that neuronal cells in the penumbra undergo ferroptotic cell death after cerebral I/R. Treatment strategies that combine with ferroptosis may improve neuronal rescue after ischaemic stroke. FtMt prevents I/R-induced ferroptosis by accurately controlling the iron-related redox balance, thus inhibiting the transformation from the penumbra to the infarct core. In addition, as a mitochondrial-localized protein, we speculate that FtMt also participates in the recovery process of neuronal cells in the penumbra by affecting mitochondrial energy metabolism. However, further research is needed to prove this point of view.

In summary, our data indicate that FtMt plays a protective role in cerebral I/R. FtMt limits I/R-induced iron overload and iron-dependent lipid peroxidation, which suppresses ferroptosis in the penumbra. Finally, our data suggest that FtMt may be a potential therapeutic target in ischaemic stroke.

## Materials and methods

### Animals

All mice were housed in pathogen-free cages under conditions controlled for temperature (22 °C) and humidity (40%) under a 12 h/12 h light/dark cycle. All animals were provided a standard rodent diet and water *ad libitum*. Three-month-old C57BL/6J wild-type male mice, *Ftmt*-knockout male mice and FtMt-overexpressing male mice were used in this study. All procedures were carried out in accordance with the National Institutes of Health Guide for the Care and Use of Laboratory Animals and were approved by the Animal Care and Use Committee of the Hebei Science and Technical Bureau in China. Randomization and blinding were used in animal experiments.

### MCAO Model

Transient MCAO was used to induce ischaemic stroke in adult mice, as reported previously^53,54^. The animals were anaesthetized with an intraperitoneal injection of chloral hydrate (3.5 mg/kg) and then placed on their backs to expose the neck area. The common carotid artery (CCA), external carotid artery (ECA), and internal carotid artery (ICA) were carefully separated from the adjacent tissue and vagus nerve via a midline neck incision. The distal ECA was tied off and opened by arteriotomy, and a nylon monofilament (602234PK10Re, Doccol Corp., CA, USA) was inserted and advanced upwards approximately 10 mm past the CCA bifurcation. Focal cerebral blood flow was measured by laser Doppler flowmetry (Perimed, Sweden) to confirm MCAO. After an occlusion period of 90 min, the monofilament was removed to commence reperfusion. Body temperature was maintained at 37 ^ο^C with a heating pad during the entire procedure. Sham-operated control mice were treated similarly, but the monofilament was not inserted. All animals were allowed access to water and food *ad libitum* after surgery. Mice that had excessive bleeding during the surgery or experienced cerebral haemorrhage, or when the filament was removed before reperfusion, were excluded from the study.

### Assessment of neurologic deficits

After 24 h of reperfusion, the signs of neurologic impairment were evaluated according to a five-point scale as previously described:^55^ 0, no observable neurologic deficit; 1, failed to extend right forepaw; 2, circled to the right; 3, fell to the right; 4, could not walk spontaneously; 5, dead. The assessment was performed by a masked investigator and then confirmed by another investigator blinded to the experimental groups.

### 2,3,5-Triphenyltetrazolium chloride (TTC) staining and infarct volume assessment

Mice were anaesthetized with chloral hydrate and sacrificed 24 h after reperfusion. The brains were immediately removed, placed at −20 °C for 15 min and then sliced into 1 mm coronal sections with a metallic brain matrix. The brain slices were incubated in 2% TTC (#1.08380, Sigma-Aldrich, USA) in phosphate buffer and stained for 10 min at 37 °C in the dark. They were then fixed in 8% paraformaldehyde at 4 °C until imaging. The unstained white area of the ipsilateral hemisphere was considered the infarcted area, whereas the non-infarcted area of the contralateral hemisphere was stained red. The infarcted and non-infarcted areas were measured in each section with ImageJ by a blinded investigator. The infarct volume was calculated by the following formula: (contralateral hemisphere volume−non-infarcted ipsilateral hemisphere volume)/contralateral hemisphere volume × 100%^56^.

### Evans blue extravasation

Disruption of the blood–brain barrier (BBB) was investigated using Evan’s blue (EB) dye (#E2129, Sigma-Aldrich, USA) as reported previously after I/R^57^. Briefly, EB dye (2% diluted in saline, 4 mL/kg) was injected into the caudal vein and allowed to circulate for 2 h. The mice were deeply anaesthetized, and saline was perfused transcardially until colourless perfusion fluid was obtained from the right atrium, after which the two hemispheres of the brain were dissected. The EB dye was extracted by homogenizing the sample in 2 mL of saline and 1.5 mL of 60% trichloroacetic acid. The mixture was vortexed for 2 min and centrifuged for 30 min at 1000 × *g*. The absorption of this supernatant was assessed at 610 nm. The content of EB dye extract from the brain is expressed as μg/g tissue and was determined based on a standard curve.

### Nissl staining

Twenty-four hours after reperfusion, mice were anaesthetized with chloral hydrate and then fixed by transcardial perfusion with saline followed by 4% paraformaldehyde. The brains were postfixed in the same solution for 4 h and cut into 15 μm sections. Coronal sections were immersed in Nissl staining solution (#C0117, Beyotime, China) for 2 min, rinsed with a series of ethanol concentrations from 50 to 100% and immersed twice in xylene for 5 min. The sections were then mounted under coverslips in DPX mounting medium and air dried.

### Measurement of brain iron and zinc

Total brain iron/zinc was determined using inductively coupled plasma mass spectrometry (ICP-MS) as previously described^58^. Samples were dried and then resuspended in 1 mL of 65% nitric acid overnight. The samples were heated for 20 min at 90 °C, and 1 mL 30% H_2_O_2_ was added for a further 20 min incubation at 70 °C followed by a 6 h incubation at 100 °C. The digested samples were then dissolved in 2 mL of ultrapure water and assayed by ICP-MS, and the concentrations determined from a standard curve were then normalized to dry tissue weight.

### Perl’s staining

Iron content was also assessed by 3,3-diaminobenzidine tetrahydrochloride (DAB)-enhanced Perl’s staining^58^. Fifteen-micrometre sections were treated with 3% H_2_O_2_ for 10 min. The slides were then immersed in Perl’s solution for 12 h. After being washed with phosphate-buffered saline (PBS), the samples were treated with DAB for 10 min to intensify the chromogenic reaction.

### Synchrotron radiation X-ray fluorescence

To determine the iron distribution in the brain after ischaemic stroke, micro-X-ray fluorescence (μ-XRF) microspectroscopy was performed. Mice were anaesthetized with chloral hydrate, and saline was perfused transcardially. Then, the brains of each mouse were immediately frozen at −20 °C for 20 min and cut into 50 μm sections. Equivalent slices from the same coordinates were fixed onto 3 mm-thick Mylar films (polycarbonate) from each of the two groups of mice. The samples were dried at room temperature before analysis. μ-XRF was conducted at the BL15U1 endstation of the Shanghai Synchrotron Radiation Facility and the 4W1B endstation of the Beijing Synchrotron Radiation Facility. The incident X-ray energy was maintained at 15 keV and focused down to 110 μm in diameter with a polycapillary lens. Two-dimensional maps were acquired by step mode: the sample was held on a precision motor-driven stage, and scanning was performed 110 μm stepwise. A Si(Li) solid-state detector was used to detect X-ray fluorescence emission lines with a live time of 1 s. The results were analyzed using Origin 8.0.

### Transmission electron microscopy

After 24 h of reperfusion, mice were anaesthetized, transcardially perfused with saline and fixed with 3% glutaraldehyde. The brains were immediately removed, and the penumbra area of the cortical tissue was cut into 1 mm × 1 mm × 1 mm pieces. The tissues were fixed in 3% glutaraldehyde overnight at 4 °C, immersed in 1% osmic acid in 0.1 M phosphate buffer for 30 min and then dehydrated and embedded in Araldite. Ultrathin sections (200 nm) were cut with an ultramicrotome, stained with uranyl acetate and lead citrate and examined using an electron microscope (H-7650, Hitachi, Tokyo, Japan).

### Mass spectrometry imaging (MSI)

After 24 h of reperfusion, mice were anaesthetized with chloral hydrate, and saline was perfused transcardially. The brain of each mouse was immediately removed and frozen at −20 °C for 20 min. Then, the frozen brain was mounted on a cryotome sample holder at −20 °C using water as the only adhesive, thus avoiding use of any frozen specimen embedding medium. The brain was cut into 10-μm-thick slices, and the slices were thaw-mounted on glass slides and placed in a −80 °C freezer until the time of analysis. On the day of analysis, the sample slides were taken directly from the freezer to a vacuum desiccator for 10 min. Then, a solution of 40 mg/mL 2,5-dihydroxybenzoic acid (DHB; in MeOH/H_2_O/TFA = 49.95/49.95/0.1, v/v/v) was sprayed on the sections with a pneumatic sprayer with a flow rate of 10 μL/min and a nitrogen gas pressure of 1 to 2 bar. After matrix application, the sample was placed in an atmospheric pressure scanning microprobe matrix-assisted laser desorption/ionization imaging source (AP-SMALDI10, TransMIT GmbH, Giessen, Germany) coupled to an orbital trapping mass spectrometer (Q Exactive, Thermo Fisher Scientific GmbH, Bremen, Germany). For analyte ionization, a solid-state laser with a wavelength of 343 nm and a frequency of 2000 Hz with a 0.5 ns pulse width was used. The pixel size was 50 μm. The sections were measured in positive ion mode with a *m/z* range of 300–1200. The mass resolution was 70,000 with the target voltage set to 4.0 kV.

### Rotarod treadmill test

The rotarod treadmill test was used to evaluate the motor coordination of the mice. The animals were first trained on the accelerating rotor mode (10 speeds from 4 to 40 rpm for 5 min) in 3 trials per day on 5 consecutive days prior to surgery. The interval between when a mouse mounted the rod to when it fell off was recorded as the latency time. Mice that lasted for 5 min on the rod during the training phase were selected for the cerebral I/R model. Performance in the rotarod test was measured three times a day in the 4 days following the surgery.

### Measurement of malondialdehyde (MDA) and glutathione (GSH)

MDA and total GSH levels in the brain were determined using commercial kits from the Beyotime Institute of Biotechnology (#S0131M, #S0053, Shanghai, China) according to the manufacturer’s instructions. The MDA content was assessed using the thiobarbituric acid (TBA) method, which is based on spectrophotometric measurement of the product of the reaction of TBA with MDA. The MDA concentrations were calculated by the absorbance of the product at 532 nm. The total GSH content was determined with an optimized enzymatic recycling method using GSH reductase. The formation of 2-nitro-5-thiobenzoic acid was analyzed at 412 nm, the absorbance at which was directly proportional to the concentration of GSH.

### Oxygen and glucose deprivation and reperfusion (OGD/R)

Cells were washed twice in deoxygenated glucose-free DMEM (oxygen and glucose deprivation [OGD] medium) and then transferred to this medium. The cells were then placed in a hypoxic chamber with 1% O_2_/5% CO_2_/94% N_2_ at 37 °C. OGD was carried out for 5 h. After that, cells were reoxygenated via the addition of normoxic glucose-containing medium and incubation for an additional 18 h under normal conditions. The cells were then collected for subsequent analysis.

### Cell lines and drug treatment

The stable FtMt-expressing SH-SY5Y cell line (FtMt-SY5Y) and a pcDNA3.1(-) empty vector-transfected cell line (vector-SY5Y) were generated as described previously^30^. Cells were authenticated by short tandem repeat (STR) profiling and tested for mycoplasma contamination. The cells were maintained in DMEM with heat-inactivated foetal calf serum (10%, vol/vol), glucose (4.5 mg/mL), penicillin (100 U/mL) and streptomycin (100 µg/mL) in a 37 °C humidified incubator with 5% CO_2_. Fer-1 (#S7243, Selleckchem, Houston, TX, USA) and deferoxamine (DFO; #D9533, Sigma-Aldrich, USA) were used in this study. For DFO treatment, the cells were incubated with different concentrations of DFO at the beginning of reperfusion. For Fer-1 treatment, the cells were preincubated with medium containing different concentrations of inhibitor for 4 h prior to OGD/R treatment; the drugs were present throughout all treatment periods.

### Primary cortical neuron culture

Dissociated cortical neurons were prepared and maintained as previously described^59^. The cortexes from postnatal day-1 wild-type or *Ftmt*-knockout mouse pups were dissected, cleaned in PBS and then digested with 0.125% trypsin. The dissociated cells were plated onto poly-L-lysine-coated dishes at a density of 10^5^ cells/cm^2^ and cultured in minimal essential medium (MEM; Invitrogen, USA) containing 10% foetal bovine serum. The cells were maintained at 37 °C in a humidified atmosphere containing 5% CO_2_ for 8 h, after which the medium was changed to neurobasal medium supplemented with B-27. The medium was changed every 3 days. After 7 days of culture, the cells were cultured under OGD conditions for 5 h and then reoxygenated for 18 h.

### MTT assay

An MTT assay was used to evaluate cell viability. After OGD/R treatment, the culture medium was removed, MTT (0.5 mg/mL) was added, and the cells were incubated at 37 °C in a 5% CO_2_ incubator for 4 h. DMSO (150 µL) was then added to dissolve the formazan, and the absorbance at 570 nm was measured using a microplate reader.

### Measurement of the intracellular labile iron pool (LIP)

Intracellular LIP levels were measured based on a method described in the literature^30^. In brief, after OGD/R treatment, cells were harvested, washed and resuspended in a buffer containing 140 mM NaCl, 5 mM KCl, 1 mM MgCl_2_, 5.6 mM glucose, 1.5 mM CaCl_2_ and 20 mM HEPES (pH 7.4). Calcein-AM was added to a final concentration of 0.25 μM. The mixture was incubated for 30 min at 37 °C, and the cells were then washed 3 times. The cells were resuspended in medium and transferred to a cuvette. The fluorescence intensity of calcein-AM (#C3099, Invitrogen) was quantified by a microplate reader (Synergy H5) at an excitation wavelength of 485 nm and an emission wavelength of 520 nm. Once the baseline fluorescence stabilized, salicylaldehyde isonicotinoyl hydrazine (SIH) was added to a final concentration of 100 μM, and the increase in fluorescence intensity reflected the level of calcein-bound iron.

### Measurement of lipid peroxidation in cells

Cells were seeded in 6-well dishes before the experiment. After OGD/R treatment, the cells were harvested by trypsinization and washed with Hanks Balanced Salt Solution (HBSS; #88284, Invitrogen). The cells were resuspended in 500 μL of HBSS containing 2 μM C11-BODIPY (581/591) (#D3861, Invitrogen) and incubated for 10 min at 37 °C in an incubator with 5% CO_2_. The cells were then washed and resuspended in 500 μL of HBSS, strained through a 40 μm cell strainer and analyzed by flow cytometry (CytoFLEX, Beckman Coulter) or microplate reader.

### RNA isolation and quantitative PCR

Total RNA was extracted from tissues using TRIzol reagent (Invitrogen) according to the manufacturer’s instructions. Total RNA (1 μg) was reverse transcribed into cDNA using a PrimeScript RT Kit (#RR047A, Takara) according to the manufacturer’s instructions. PCR amplification was performed with SYBR Green PCR Master Mix (#A301-01, GenStar) via a Bio-Rad CFX Connect Real-Time System with the following cycling parameters: 95 °C for 10 min followed by 40 cycles of 95 °C for 15 s and then 60 °C for 30 s. β-Actin was used as a housekeeping gene control. The primer sequences used are provided in supplementary table 1.

### Immunofluorescence

Tissue slices were washed three times with PBS. Antigen retrieval was performed in a microwave oven for 10 min in 10 mM citrate buffer (pH 6.0). After blocking for 1 h with normal goat serum prepared in PBS, the slices were incubated overnight at 4 °C with a mouse anti-Iba1 monoclonal antibody (1:200; #MABN92, Millipore Corporation, Temecula, CA) or a rabbit anti-4-hydroxynonenal (4-HNE) polyclonal antibody (1:400; #HNE13-M, Alpha Diagnostic International, San Antonio, TX, USA). The slides were then washed three times for 5 min with PBS. The following secondary antibodies were used for 50 min incubations at 37 °C: DyLight 549-conjugated goat anti‐rabbit IgG (1:200; #A23320, Abbkine Scientific Co., Ltd., Wuhan, China) and DyLight 488-conjugated goat anti‐mouse IgG (1:200; #A23210, Abbkine Scientific Co., Ltd., Wuhan, China). After washing and mounting, the sections were analyzed with an Olympus FV3000 confocal laser scanning microscope.

### Western blot analysis

Protein expression was assessed by Western blot analysis as previously described^31^. The following antibodies were used: anti-FtMt (1:5000) was a kind gift from Prof Sonia Levi, Italy; anti-ferroportin1 (FPN1) (1:5000) and anti-divalent metal transporter 1 (DMT1 ± IRE) (1:5000) were obtained from Alpha Diagnostic International (#MTP11-S, #NRAMP21-S, #NRAMP23-S, San Antonio, TX, USA); anti-β-actin (1:10000), and anti-transferrin receptor 1 (TfR1) (1:2000) was obtained from Sigma-Aldrich (#A5441, #SAB4200398, St. Louis, MO, USA); anti-STAT3 (1:2000), anti-phosphorylated STAT3 (P-STAT3) (1: 2000), anti-ferritin light chain (FtL) (1:10000), and anti-FtH (1:10000) were obtained from Abcam Trading [Shanghai] Company Ltd. (#ab68153, #ab76315, #ab109373, #ab183781); anti-12/15-lipoxygenase (LOX) (1:500, #sc-133085) was obtained from Santa Cruz Biotechnology; anti-TNFa (1:5000, #60291-1-lg) and anti-IL6 (1:2000, #21865-1-AP) were obtained from Proteintech, Wuhan, China. The immunoreactive proteins were detected using the enhanced chemiluminescence (ECL) method and quantified by transmittance densitometry using ImageJ software.

### Statistical analysis

All experiments were performed at least three times. The number of samples per group is indicated in the corresponding figure legends as n. All data are presented as the mean ± SEM. Data were analyzed using GraphPad Prism-6 and SPSS 16.0. All data were tested for normality distribution. Comparisons between two groups were made by Student’s *t* test (two-tailed). For multi-group comparisons, one-way ANOVA with Tukey’s post hoc test was used. Nonparametric analyses were performed with the Kruskal–Wallis H test (multi-group) or Mann–Whitney U test (two groups). Differences were considered statistically significant when the *P*-value was < 0.05.

## Supplementary information

supplementary information

## Data Availability

The datasets used and analyzed during the current study are available from the corresponding author on reasonable request. All data generated or analyzed during this study are included in this published article and its supplementary information files. Data sharing is not applicable to this article as no datasets were generated or analyzed during the current study.
