# Supplementary material for: Mitochondrial ferritin attenuates cerebral ischaemia/reperfusion injury by inhibiting ferroptosis
Source: Cell Death Dis. 2021 May 5;12(5):447. doi: 10.1038/s41419-021-03725-5 (PMC8099895; doi:10.1038/s41419-021-03725-5)
Supplement: Supplementary file 1 — supplementary information [file 41419_2021_3725_MOESM1_ESM.docx]

**Supplementary Information**

Mitochondrial ferritin attenuates cerebral ischaemia/reperfusion injury by inhibiting ferroptosis

Peina Wang^a,^, Yanmei Cui^a^, Qianqian Ren^a^, Bingqi Yan^a^, Yashuo Zhao^a,c^, Peng Yu^a^, Guofen Gao^a^, Honglian Shi^b^, Shiyang Chang ^d,*^, Yan-Zhong Chang^a,*^

**Supplementary table1 Primer sequences used for real-time RT-PCR analysis.**

| **Gene** | **Forward (5’-3’)** | **Reverse (5’-3’)** |
| --- | --- | --- |
| *Ftmt* | AGC ACA TCA GCT CTG CAC TG | AGG CCA GTA GGG GAC CTA AA |
| *Actb* | AGG CCC AGA GCA AGA GAG GTA | TCT CCA TGT CGT CCC AGT TG |
| *Ptgs2* | CTGCGCCTTTTCAAGGATGG | GGGGATACACCTCTCCACCA |
| *Hamp* | TTGCGATACC AATGCAGAAGAG | AATTGTTACAGCATTTACAGCAGAAGA |
| *TNFα* | CCTGTAGCCCACGTCGTAG | GGGAGTAGACAAGGTACAACCC |
| *IL1β* | GAAATGCCACCTTTTGACAGTG | TGGATGCTCTCATCAGGACAG |
| *IL-6* | GCTACCAAACTGGATATAATCAGGA | CCAGGTAGCTATGGTACTCCAGAA |


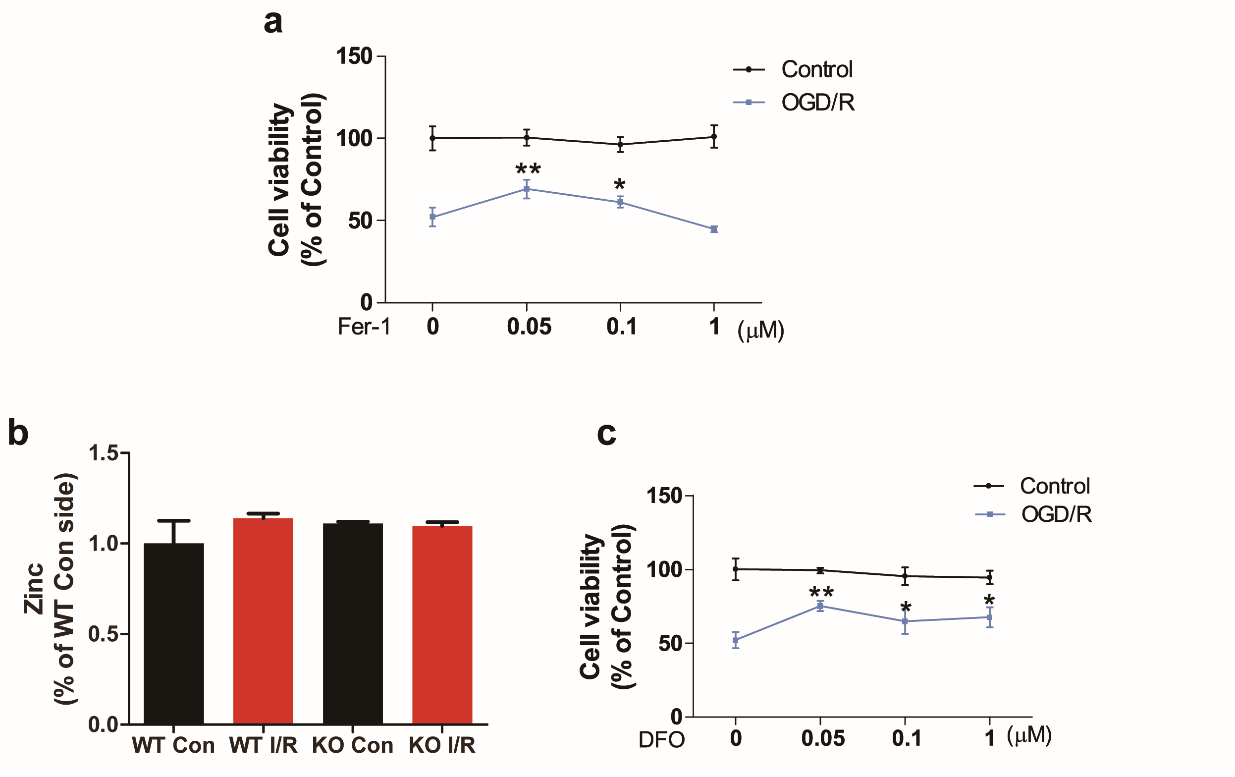


**Supplementary Figure 1 The effects of DFO and Fer-1 on OGD/R-induced injury in** **SH-SY5Y cells and zinc content in the brains of mice after ischaemic stroke** (A) SH-SY5Y cells were pre-treated for 4 h with the indicated concentrations of Fer-1 and then subjected to OGD/R treatment with Fer-1. Cell viability was measured using an MTT assay (n=3). (B) Zinc content as determined by ICP-MS in wild-type and *Ftmt*-knockout mice 24 h after MCAO/reperfusion (n=6). (C) SH-SY5Y cells were subjected to OGD treatment for 5 h and then exposed to normoxic glucose-containing medium containing the indicated concentrations of DFO for another 18 h. Cell viability was determined using an MTT assay (n=3). The results are presented as the mean ± SEM. ^*/$^*P* < 0.05, ^**/$$^*P* < 0.01.
